# Association between CT imaging features and KIT mutations in small intestinal gastrointestinal stromal tumors

**DOI:** 10.1038/s41598-019-43659-9

**Published:** 2019-05-10

**Authors:** Yi-qiong Yin, Chun-juan Liu, Bo Zhang, Yue Wen, Yuan Yin

**Affiliations:** Department of Gastrointestinal Surgery, West China Hospital, Sichuan University, 37# Guo Xue Xiang, Chengdu, Sichuan 610041 China

**Keywords:** Gastrointestinal cancer, Cancer imaging

## Abstract

Small intestinal gastrointestinal stromal tumors (GISTs) have different clinical outcomes when KIT mutations are in exons 11 or 9, which are also the most common sites of neoplastic KIT mutations. The purpose of this study is to evaluate the CT imaging features in those two groups. A total of 35 patients were enrolled, and both quantitative and qualitative CT imaging features were compared between patient groups with KIT exon 9 mutations (KIT–9) and exon 11 mutations (KIT–11). The KIT–9 group was statistically associated with a tumor size larger than 10 cm and a higher enhancement ratio when compared with those of the KIT–11 group (both *P* < 0.05). For the enhancement ratio, the receiver operating characteristic curve indicated a cut-off value of 1.60 to differentiate KIT–9 from KIT–11 tumors. Additionally, tumor necrosis was more commonly seen in the KIT-9 group. In multivariate analysis, tumor size (β = 0.206; *P* = 0.022) and KIT–9 (β = 0.389; *P* = 0.006) were independent factors associated with tumor necrosis. Taken together, KIT–9 mutant tumors tended to have CT imaging features indicative of more aggressive neoplasms. These findings may be helpful in identifying more aggressive small intestinal GISTs and optimizing treatment.

## Introduction

Gastrointestinal stromal tumors (GISTs) are the main mesenchymal tumors of the gastrointestinal tract, with an annual incidence of 10–15 cases per million^1,2^. GISTs derive from the intestinal pacemaker cells of Cajal and range in potential malignancy from indolent tumors to rapidly progressing cancer^3^. More than 80% of GISTs express KIT-activating mutations, most frequently in exon 11. This is the genetic locus that responds to tyrosine kinase inhibitor (TKI) therapy. TKI therapy provides substantial improvement in survival, particularly for patients with advanced disease and a high risk of recurrence^4^.

The second most common type of GISTs is small intestinal GISTs. They have more aggressive behavior and a poorer prognosis than gastric GISTs, partially because of their different distribution of genotypes and mutation rates. Data from previous research show that KIT exon 9 mutations are more frequent in small intestinal GISTs^5,6^. To the best of our knowledge, although several studies have described the CT imaging features of small intestine GISTs^7,8^, whether there are differences between GISTs in patients with KIT exon 9 (KIT–9) versus exon 11 (KIT–11) mutations remains unclear. Therefore, in this study, we sought to identify CT-visible differences between these groups under the hypothesis that differences in KIT activity related to the mutation site would create such features. If established, CT imaging could be a useful tool for identifying more aggressive tumors and optimizing clinical treatment.

## Results

### Baseline characteristics

The clinical and demographic details of the KIT–11 and KIT–9 groups are provided in Table 1. There were no significant differences in age, gender, and risk classification (all *P* > 0.05). The KIT–9 group was symptomatic more often than KIT–11 group (100% vs. 66.7%; *P* < 0.05). In immunohistochemical analysis, Ki-67 expression was higher in the KIT–9 group than in the KIT–11 group (9.30 ± 7.01 vs. 7.30 ± 3.88; *P* < 0.05), whereas no differences were found in CD117, Dog-1, CD34, S-100, and SMA expression (all *P* > 0.05). Upon classifying the analyzed tumors by mutation (Table 2), various disease-causing subtypes were found. Of note, codon A502_Y503dup was most commonly seen in the KIT–9 group (7/11, 63.6%), whereas codon 557_558del was most commonly seen in the KIT–11 group (11/24, 45.8%).

**Table 1:** Baseline characteristics.

	KIT–11 (n = 24)	KIT–9 (n = 11)
Age, year	54.75 ± 13.57	53.27 ± 12.07
Male, n (%)	19 (79.2%)	7 (63.6%)
Symptomatic at presentation, n (%)	16 (66.7%)	11 (100%)*
Risk classification, n (%)		
Low risk	5 (20.8%)	1 (9.1%)
Intermediate risk	3 (12.5%)	2 (18.2%)
High risk	16 (66.7%)	8 (72.7%)
Immunohistochemical analysis		
CD117, n (%)	24 (100%)	11 (100%)
Dog-1, n (%)	24 (100%)	11 (100%)
CD34, n (%)	13 (54.2%)	8 (72.7%)
S-100, n (%)	1 (4.2%)	1 (9.1%)
SMA, n (%)	11 (45.8%)	4 (36.4%)
Ki-67, %	7.30 ± 3.88	9.30 ± 7.01*

Values are presented as mean ± standard deviation or numbers (percentages).

*Means *P* < 0.05 versus KIT–11 group.

**Table 2:** Classifications of mutational profiles.

Gene	Disease causing variants
KIT –9	**Codon A502_Y503dup** • 9 (A502_Y503dup), (n = 7) **Non-codon A502_Y503dup** • 9 (Y503_F504insAY), (n = 2) • 9 (Y503_F504insAH) (n = 2)
KIT –11	**Codon 557_558del** • 11 (W557_K558delinsS), (n = 10) **Non- codon 557_558del** • 11 (D579_H580 insert IDPTQLPYD), (n = 3) • 11 (P551_W557del), (n = 2) • 11 (E554_K558del), (n = 2) • 11 (K558_I563del), (n = 2) • 11 (Y568_L576delinsCV), (n = 1) • 11 (P551_V560del,insL), (n = 1) • 11 (Q556_I571del), (n = 1) • 11 (L576P), (n = 1) • 11 (V559D), (n = 1)

### Association between CT imaging features and KIT mutations

**Figure 1:**
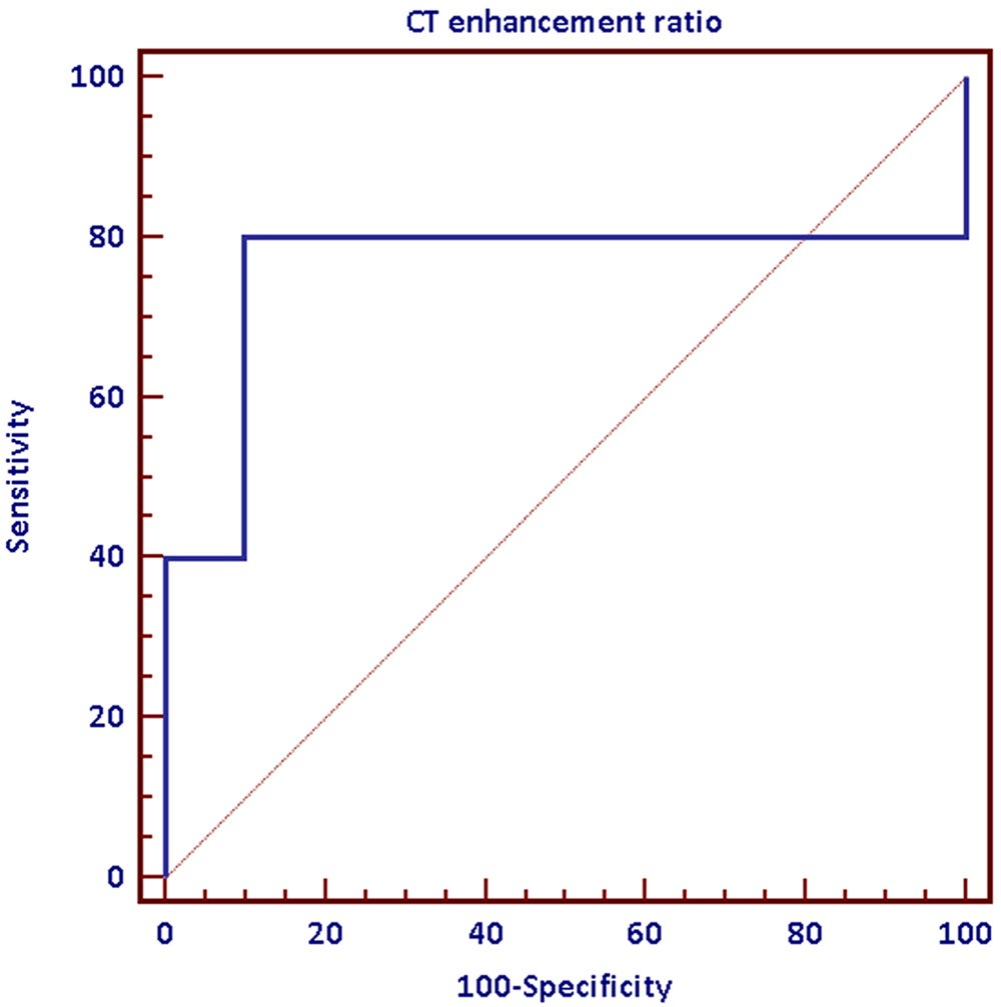
Receiver operating characteristic curve of CT enhancement ratios for differentiating tumors between KIT exon 11 and exon 9 mutations.

As shown in Table 3, tumors with exon 9 mutations were statistically associated with a tumor size larger than 10 cm (6/11, 54.5% vs. 4/24, 16.7%; *P* < 0.05) and a higher enhancement ratio on CT (1.76 ± 0.63 vs. 1.39 ± 0.28; *P* < 0.05) more often than exon 11 mutation tumors. In analyzing the tumor enhancement ratio, the receiver operator characteristic (ROC) curve produced a cut-off value of 1.60 in differentiating tumors between the two groups (sensitivity, 86.7%; specificity, 98.5%; and area under the ROC curve, 0.76) (Fig. 1). Notably, necrosis in the tumors was more commonly seen those with exon 9 mutations, although the statistical threshold was not reached. We also found that enlarged vessels feeding or draining the mass (EVFDM) seemed to appear in tumors with a more marked degree of enhancement.

**Table 3:** CT imaging features.

	KIT–11 (n = 24)	KIT–9 (n = 11)
Location, n (%)		
Jejunum	15 (62.5%)	7 (63.6%)
Ileum	9 (37.5%)	4 (36.4%)
Shape, n (%)		
Regular	7 (29.2%)	3 (27.3%)
Irregular	17 (70.8%)	8 (72.3%)
Size, n (%)		
<5 cm	6 (25.0%)	3 (27.3%)
5–10 cm	14 (58.3%)	2 (18.2%)*
>10 cm	4 (16.7%)	6 (54.5%)*
Growth pattern, n (%)		
Exophytic	10 (41.7%)	4 (36.4%)
Endophytic	2 (8.3%)	1 (9.1%)
Mixed	12 (50.0%)	6 (54.5%)
Necrosis, n (%)	11 (45.8%)	8 (72.3%)
Calcification, n (%)	5 (20.8%)	1 (9.1%)
Enhancement pattern, n (%)		
Homogenous	5 (20.8%)	2 (18.2%)
Heterogenous	19 (79.2%)	9 (81.8%)
Enhancement degree, CT unit	88.95 ± 19.90	90.22 ± 30.41
Enhancement ratio	1.39 ± 0.28	1.76 ± 0.63*
EVFDM, n (%)	6 (25.0%)	3 (27.3%)
Metastasis, n (%)	3 (12.5%)	2 (18.2%)

Values are presented as mean ± standard deviation or numbers (percentages).

*Means *P* < 0.05 versus KIT–11 group.

Abbreviations: EVFDM, enlarged vessels feeding or draining the mass.

Risk analysis for KIT mutation showed a relative risk of tumor size (5–10 and >10 cm), degree of enhancement on CT imaging, and necrosis in the tumors ranging from 1.55 to 3.27 (Table 4). In multivariate analysis, tumor size (β = 0.206; *P* = 0.022) and exon 9 mutations (β = 0.389; *P* = 0.006) were independent factors associated with tumor necrosis (Table 5).

**Table 4:** Risk analysis for KIT mutation.

	Relative risk	CI
Location		
Jejunum	0.98	0.57–1.69
Ileum	1.03	0.40–2.63
Irregular shape	0.97	0.62–1.52
Size		
5–10 cm	3.21	0.88–11.75
>10 cm	3.27	1.15–9.30
Growth pattern		
Exophytic	1.14	0.46–2.86
Endophytic	0.92	0.09–9.07
Mixed	0.92	0.47–1.79
Necrosis	1.58	0.90–2.79
Calcification	2.29	0.30–17.36
Heterogenous enhancement	0.97	0.68–1.37
Enhancement degree	1.55	0.83–2.90
EVFDM	0.91	0.28–3.01
Metastasis	0.69	0.13–3.54

Abbreviations: EVFDM, enlarged vessels feeding or draining the mass. CI, confidence interval.

**Table 5:** The risk factors of tumor necrosis by logistic regression analysis.

	Univariable analysis		Multivariable analysis	
	R	*P* value	β	*P* value
Sex	0.187	0.283	—	—
Age	−0.040	0.817	—	—
Location	0.050	0.774	—	—
Shape	0.164	0.347	—	—
Size	0.425	0.011	0.206	0.022
Growth pattern	0.087	0.619	—	—
Enhancement pattern	0.024	0.891	—	—
Enhancement degree	−0.211	0.537	—	—
Enhancement ratio	0.043	0.805	—	—
EVFDM	−0.047	0.789	—	—
Metastasis	0.321	0.060	—	—
Exon 9-mutation	0.578	<0.001	0.389	0.006

Abbreviations: EVFDM, enlarged vessels feeding or draining the mass.

## Discussion

Exon 11 and 9 mutations are the most common mutation sites of GISTs^3,4^. Our study demonstrated that the tumors with exon 9 mutations tended to be larger than those with exon 11 mutations, which is consistent with a previous study^5^. It is known that large tumor size is indicative of high risk stratification and a poor outcome^2^. As the tumor grows, secondary changes and associated complications may arise, such as bleeding, rupture, and bowel obstruction; this is a common presentation at the point at which a patient is referred to a hospital and may explain why all of the KIT–9 group patients complained of abdominal symptoms. More importantly, we found that the exon 9 subset seems to be accompanied by a higher enhancement ratio. CT enhancement mainly reflects the distribution of intratumoral vasculature. This finding may indicate that exon 9 mutant tumors are prone to hypervascularization, which relates to a more aggressive growth pattern than the less-vascularized exon 11 mutant tumors. In addition, these findings also support the results of Antonescu *et al*., who found that tumors with exon 9 mutations are associated with unfavorable clinical outcomes^5^. Our ROC analysis demonstrated that the CT enhancement ratio may be a useful tool for differentiating KIT exon 9 and exon 11 mutant tumors. Combined with pretreatment biopsy, it could greatly aid in clinical decision-making.

In addition, necrosis was more frequently seen among the KIT–9 group, although statistical significance was not reached. Upon multivariate analysis, we found that tumor size and exon 9 mutations were independent factors associated with tumor necrosis. This finding is likely to be evidence of a heterogeneous blood supply and high malignancy potential in tumors with exon 9 mutations^9^.

In our study, small intestinal GISTs occurred more often in the jejunum than in the ileum regardless of genotype, as was found in prior research on a large sample^10^. Several studies reported that ileal tumors tended to be larger than jejunal tumors, but the difference did not reach statistical significance in any of those studies^7,8,10^. GISTs often have exophytic or mixed growth patterns since they arise from the intestinal Cajal cells in the deep muscularis^11^. In this study, both subsets presented with an exophytic or a mixed growth pattern. Calcification was seen in 20.8% of the tumors with exon 11 mutations, which is similar to a report by Baheti *et al*.^7^. However, calcification seemed relatively rare in the tumors with exon 9 mutations. EVFDM was found to be similar in both groups and was more easily seen during the arterial phase. Zhou *et al*. regarded EVFDM as a predictor for risk stratification^12^, although the exact mechanism underlying this relationship is unclear and needs further study^13,14^.

Our study had several limitations. First, this was a retrospective study with a single-center population, and thus, a limited sample size and selection bias was unavoidable. Second, this was an initial, discovery-phase study. Therefore, further studies with larger samples should be performed to validate our data in a more generalized population. Third, the types of GISTs were restricted, so wild-type KIT, secondary KIT (e.g., exons 13, 14, and 17), and platelet-derived growth factor receptor α (PDGFRA) mutations were not included because of their small proportions.

In summary, this study demonstrated that GISTs with exon 9 mutations tend to have more aggressive CT imaging features, such as a larger size and higher enhancement ratio; there were also more occurrences of necrosis. These findings may be helpful in identifying more aggressive intestinal GISTs so as to optimize treatment for patients.

## Materials and Methods

### Ethics statement

This study involving human participants was approved by the Institutional Review Board of West China Hospital and performed in accordance with the Declaration of Helsinki (2000 Edition) and relevant medical research rules of China. All patient-sensitive information was treated with full confidentiality and used solely for the purpose of this study. Owing to the retrospective nature of the study and lack of identifying information, informed consent was waived.

### Study population

**Figure 2:**
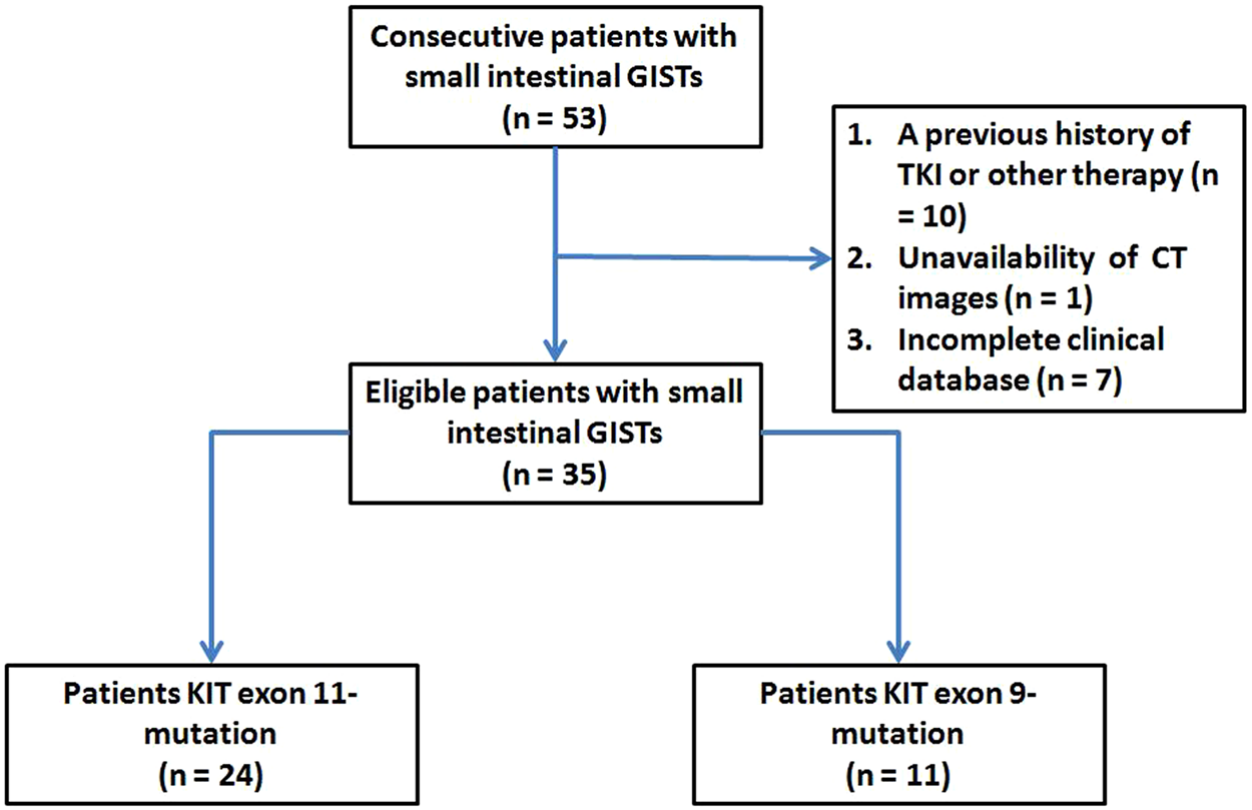
Flowchart of the study.

We included records from an electronic clinical database (January 2012 to December 2015) to identify patients with biopsy-proven, small intestine GISTs. There were 53 consecutive patients initially included. The exclusion criteria were as follows: (a) previous history of TKI therapy or other therapy, (b) unavailability of pretreatment contrast-enhanced CT images, and (c) incomplete clinical database. After applying these criteria, a total of 35 patients (24 patients with exon 11 mutations and 11 patients with exon 9 mutations) were enrolled in this study for analysis (Fig. 2).

### CT protocol

Each patient was asked to empty their bowels and drink 500–1000 mL of water within 45 min before examination. Scanning was performed using a dual-source CT scanner (Somatom Definition; Siemens Medical Solutions, Forchheim, Germany) or a 64-detector row CT scanner (Brilliance 64, Philips Medical System, Eindhoven, the Netherlands). The parameters for the former were as follows: a tube voltage of 120 kV, tube current of 200 mAs, gantry rotation time of 0.5 s, pitch of 0.9, thickness of 0.5 mm, and gap of 0.2 mm. The parameters for the latter were as follows: a tube voltage of 120 kV, tube current of 145 mAs, gantry rotation time of 0.42 s, pitch of 0.9, thickness of 0.5 mm, and gap of 0.2 mm. A total of 80–85 mL of contrast agent (Iohexol, 300 mg/mL, Beijing Beilu Pharmaceuticals, Beijing, China) was given at a flow rate of 2 mL/s via the antecubital vein, followed by 20 mL of saline solution at the same flow rate. Arterial and portal venous phases were triggered following delays of 30 and 70 s, respectively, after the administration of the contrast agent.

### Image analysis

**Figure 3:**
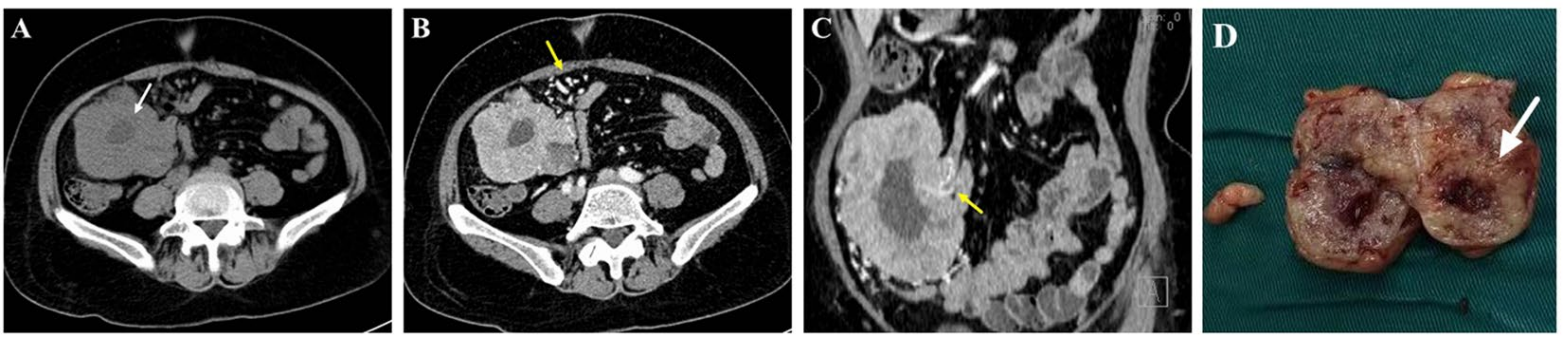
A 56-year-old woman with a small intestinal gastrointestinal stromal tumor. An axial non-enhanced CT image (**A**) reveals the necrosis in the tumor (white arrow). An axial contrast-enhanced CT and a coronal reconstructed image (**B**,**C**) reveal some enlarged vessels feeding or draining the mass (yellow arrow). Pathological analysis of the specimen demonstrated necrosis in the tumor (white arrow) that corresponded to the area in CT imaging.

Two clinicians (five experiences in abdominal imaging) who were blind to the patient group (KIT–9/KIT–11) analyzed these images independently on a workstation (Syngo; Siemens Medical System, Forchheim, Germany). The CT imaging features of the tumors included the primary location, shape, size, growth pattern, necrosis, calcification, enhancement pattern/degree, enhancement ratio, metastasis, and EVFDM (Fig. 3). The shape of the tumor was classified as regular or irregular. The size of the tumor was divided into three groups (<5, 5–10, and > 10 cm) according to the largest dimension. The growth pattern was classified as exophytic, endophytic, and mixed. Necrosis was defined as non-enhanced regions of the tumor at the portal venous phase. The enhancement pattern was classified as homogeneous or heterogeneous by eye at the portal venous phase. The enhancement degree was determined by measuring the CT units in tumor parenchyma at the portal venous phase while avoiding the necrotic regions. The enhancement ratio was defined as the CT unit ratio of tumor parenchyma divided by that of the erector spinae muscle at the same level^8,12,14^.

### Statistical analysis

The data were analyzed using SPSS software (version 17.0 for Windows, SPSS, Chicago, IL, USA). A D’Agostino–Pearson normality test was used to check the normality of data. Continuous data were presented as mean ± standard deviation or median (interquartile range), and categorical data were presented as numbers and percentages. Student’s t-tests or Mann–Whitney U-tests were performed to assess the differences for quantitative CT features between the two mutation groups. χ^2^ or Fisher exact tests were performed to assess the differences of qualitative CT features between the two mutation groups. A ROC curve was used to predict the sensitivity and specificity of the CT enhancement ratio in differentiating between the two groups. The relative risks for KIT mutation were determined for each CT feature. Binary logistic regression was performed to identify any association with tumor necrosis. A *P* value less than 0.05 was considered statistically significant.

## Data Availability

The datasets generated and analyzed during the current study are available from the corresponding author on reasonable request.
